# Supplementary material for: Anxiety and associated factors among medical and surgical patients in Ethiopia: A systematic review and meta-analysis
Source: PLoS One. 2024 Jul 24;19(7):e0306413. doi: 10.1371/journal.pone.0306413 (PMC11268606; doi:10.1371/journal.pone.0306413)
Supplement: S3 File — (DOCX) [file pone.0306413.s003.docx]

**Table 1**. Quality assessments of anxiety and associated factors among medical and surgical patients in Ethiopia (selected studies in this meta-analysis and systematic review).

| **Author’s name, year of**  **Publication** | **Q1** | **Q2** | **Q3** | **Q4** | **Q5** | **Q6** | **Q7** | **Q8** | **Q9** | **Total score (9%)** |
| --- | --- | --- | --- | --- | --- | --- | --- | --- | --- | --- |
| N. Afrassa et al., 2022 | Y | Y | NA | NR | Y | Y | Y | Y | Y | 7 |
| Bedaso and Ayalew, 2019 | Y | Y | Y | Y | Y | Y | Y | Y | Y | 9 |
| Y.A. Ferede et al., 2022 | Y | Y | Y | Y | Y | Y | Y | Y | Y | 9 |
| Mulugeta et al, 2018 | Y | Y | NA | NR | Y | Y | Y | Y | Y | 7 |
| Nigussie et al., 2014 | Y | Y | NA | Y | Y | Y | Y | Y | Y | 8 |
| Shewangzaw Engda et al., 2022 | Y | Y | NA | NR | Y | Y | Y | Y | Y | 7 |
| Srahbzu et al., 2018 | Y | Y | Y | NR | Y | Y | Y | Y | Y | 8 |
| Woldegerima, YB et al., 2018 | NA | Y | NA | Y | Y | Y | Y | Y | Y | 7 |
| Wondmieneh and adam, 2020 | Y | Y | NA | Y | Y | Y | Y | Y | Y | 8 |
| Aberha M et al., 2016 | Y | Y | Y | Y | Y | Y | Y | Y | Y | 9 |
| Bantalem T.A. et al., 2022 | Y | Y | NA | Y | Y | Y | Y | Y | Y | 8 |
| Ayalew M, et al., 2022 | Y | Y | Y | Y | Y | Y | Y | Y | Y | 9 |
| Amsalu Belete et al., 2014 | Y | Y | Y | Y | Y | Y | Y | Y | Y | 9 |
| Duko et al., 2019 | Y | Y | Y | NR | Y | Y | Y | Y | Y | 8 |
| Edmealem and Sanchez Olis, 2020 | Y | Y | Y | Y | Y | Y | Y | Y | Y | 9 |
| Endeshaw D, et al., 2022 | Y | Y | Y | Y | Y | Y | Y | Y | Y | 9 |
| Hajure et al., 2020 | Y | Y | Y | NR | Y | Y | Y | Y | Y | 8 |
| Nigussie et al., 2023 | Y | Y | Y | Y | Y | Y | Y | Y | Y | 9 |
| Tesfaw et al., 2016 | Y | Y | Y | NR | Y | Y | Y | Y | Y | 8 |
| Tesfaw et al. 2022 | Y | Y | Y | Y | Y | Y | Y | Y | Y | 9 |
| N. S. Tibebu et al., 2023 | Y | Y | Y | NR | Y | Y | Y | Y | Y | 8 |
| Tiki T et al., 2017 | Y | Y | Y | NR | Y | Y | Y | Y | Y | 8 |
| Yousuf, A et al.,2020 | Y | Y | Y | Y | Y | Y | Y | Y | Y | 9 |

**Key:** **Y**= Yes; **NR**= Not reported, **NA**=Not appropriate

**Question codes:**

1. Was the sample frame appropriate to address the target population?

2. Were study participants sampled in an appropriate way?

3. Was the sample size adequate?

4. Were the study subjects and the setting described in detail?

5. Was the data analysis conducted with sufficient coverage of the identified sample?

6. Were valid methods used for the identification of the condition?

7. Was the condition measured in a standard, reliable way for all participants?

8. Was there appropriate statistical analysis?

9. was the response rate adequate, and if not, was the low response rate managed appropriately?
