# Supplementary material for: Anxiety and associated factors among medical and surgical patients in Ethiopia: A systematic review and meta-analysis
Source: PLoS One. 2024 Jul 24;19(7):e0306413. doi: 10.1371/journal.pone.0306413 (PMC11268606; doi:10.1371/journal.pone.0306413)
Supplement: Attachment — Submitted filename: PONE-D-23-22543.pdf [file pone.0306413.s004.pdf]

PLOS ONE

Anxiety and associated factors among medical and surgical patients in Ethiopia: A systematic review and meta-analysis

--Manuscript Draft--

**Manuscript Number:**	PONE-D-23-22543
**Article Type:**	Research Article
**Full Title:**	Anxiety and associated factors among medical and surgical patients in Ethiopia: A systematic review and meta-analysis
**Short Title:**	Anxiety among medical and surgical patients in Ethiopia
**Corresponding Author:**	Gidey Rtbey, MSc<br>University of Gondar College of Medicine and Health Sciences<br>GONDAR, ETHIOPIA
**Keywords:**	anxiety; medical patients; preoperative surgical patients; meta-analysis; Ethiopia
**Abstract:**	Background<br><br>Anxiety, in addition to being a natural emotional response to risk, it can also have physical and psychological components. Patients diagnosed with chronic medical illness and patients appointed to undergo surgical procedures face various degrees of anxiety due to doubts related to the outcome of surgery, and the socioeconomic and psychological costs of the medical illness. This can have a negative impact on achieving the ultimate goals of managing the health problem and decreasing their quality of life. In Ethiopia even though different studies were conducted on anxiety and associated factors both among medical and surgical patients, the findings were found to be inconsistent and had a wide discrepancy. So, this systematic review and meta-analysis revealed the estimated pooled effect size of anxiety among this population and guides to plan appropriate intervention at a national level.<br><br>Methods<br><br>Observational studies conducted on anxiety and associated factors among medical and surgical patients in Ethiopia were included based on the criteria after independent selection by two authors. Data were extracted using a Microsoft Excel spreadsheet and then exported to STATA version-11 for analysis. The random-effect model was used to estimate the pooled effect size of anxiety and its determinants from the selected studies with 95% confidence intervals. Funnel plots analysis and Egger regression tests were employed to check the presence of publication bias. Sub-group analysis and sensitivity analysis were also employed.<br><br>Result<br><br>A total of 8490 study participants were included from the twenty-three selected studies in this meta-analysis and systematic review. The pooled prevalence of anxiety among medical and surgical patients in Ethiopia was 48.82% with 95 CI (42.66, 54.99). The subgroup analysis revealed that anxiety was highest in Oromia region 54.11%, 95% CI (40.69, 54.99), among surgical patients 56.97%, 95% CI (49.79, 64.15), and studies that used the S-STAI tool 57.55%, 95% CI (51.30, 63.79). Being female [OR=2.84(2.02, 4.01)], fear of death [OR=2.93(1.57, 5.50)], and history of previous surgery[AOR=0.42(0.27, 0.065)] among surgical patients and being female [AOR= 2.35(1.94, 2.850] having poor social support [OR=2.22(1.62, 3.05)], perceived stigma [OR=4.25(1.97, 9.18)] and family history of mental illness [AOR=1.86(1.21, 2.86)] among medical patients were significantly associated with anxiety in this systematic review and meta-analysis.<br><br>Conclusion and recommendation<br><br>The pooled prevalence of anxiety in this systematic review and meta-analysis in Ethiopia was found to be high among medical and surgical patients. Therefore, it would be good for health care professionals to assess patients for anxiety in addition to

Powered by Editorial Manager® and ProduXion Manager® from Aries Systems Corporation

	managing their medical or surgical cases in order to early detection and intervene in them.
**Order of Authors:**	Gidey Rtbey, MSc
	Milen Mihertabe
	Fantahun Andualem, MSc
	Mamaru Melkam
	Setegn Fentahun, MSc
**Additional Information:**	
**Question**	**Response**
**Financial Disclosure**<br><br>Enter a financial disclosure statement that describes the sources of funding for the work included in this submission. Review the submission guidelines for detailed requirements. View published research articles from *PLOS ONE* for specific examples.<br><br>This statement is required for submission and **will appear in the published article** if the submission is accepted. Please make sure it is accurate.<br><br>**Unfunded studies**<br>Enter: *The author(s) received no specific funding for this work.*<br><br>**Funded studies**<br>Enter a statement with the following details:<br>• Initials of the authors who received each award<br>• Grant numbers awarded to each author<br>• The full name of each funder<br>• URL of each funder website<br>• Did the sponsors or funders play any role in the study design, data collection and analysis, decision to publish, or preparation of the manuscript?<br>• **NO** - Include this sentence at the end of your statement: *The funders had no role in study design, data collection and analysis, decision to publish, or preparation of the manuscript.*<br>• **YES** - Specify the role(s) played.<br><br>* typeset	The author(s) received no specific funding for this work.
**Competing Interests**<br><br>Use the instructions below to enter a	The authors have declared that no competing interests exist.

competing interest statement for this submission. On behalf of all authors, disclose any competing interests that could be perceived to bias this work—acknowledging all financial support and any other relevant financial or non-financial competing interests.<br><br>This statement is required for submission and **will appear in the published article** if the submission is accepted. Please make sure it is accurate and that any funding sources listed in your Funding Information later in the submission form are also declared in your Financial Disclosure statement.<br><br>View published research articles from *PLOS ONE* for specific examples.<br><br>**NO authors have competing interests**<br><br>Enter: *The authors have declared that no competing interests exist.*<br><br>**Authors with competing interests**<br><br>Enter competing interest details beginning with this statement:<br><br>*I have read the journal's policy and the authors of this manuscript have the following competing interests: [insert competing interests here]*<br><br>* typeset	
**Ethics Statement**<br><br>Enter an ethics statement for this submission. This statement is required if the study involved:<br><br>• Human participants<br>• Human specimens or tissue<br>• Vertebrate animals or cephalopods<br>• Vertebrate embryos or tissues<br>• Field research	since this study is systematic review and meta-analysis no ethical review was needed.

Write "N/A" if the submission does not require an ethics statement.

General guidance is provided below. Consult the submission guidelines for detailed instructions. **Make sure that all information entered here is included in the Methods section of the manuscript.**

Format for specific study types

Human Subject Research (involving human participants and/or tissue)

- Give the name of the institutional review board or ethics committee that approved the study
- Include the approval number and/or a statement indicating approval of this research
- Indicate the form of consent obtained (written/oral) or the reason that consent was not obtained (e.g. the data were analyzed anonymously)

Animal Research (involving vertebrate animals, embryos or tissues)

- Provide the name of the Institutional Animal Care and Use Committee (IACUC) or other relevant ethics board that reviewed the study protocol, and indicate whether they approved this research or granted a formal waiver of ethical approval
- Include an approval number if one was obtained
- If the study involved *non-human primates*, add *additional details* about animal welfare and steps taken to ameliorate suffering
- If anesthesia, euthanasia, or any kind of animal sacrifice is part of the study, include briefly which substances and/or methods were applied

Field Research

Include the following details if this study involves the collection of plant, animal, or other materials from a natural setting:

- Field permit number
- Name of the institution or relevant body that granted permission

Powered by Editorial Manager® and ProduXion Manager® from Aries Systems Corporation

**Data Availability**<br><br>Authors are required to make all data underlying the findings described fully available, without restriction, and from the time of publication. PLOS allows rare exceptions to address legal and ethical concerns. See the PLOS Data Policy and FAQ for detailed information.<br><br>A Data Availability Statement describing where the data can be found is required at submission. Your answers to this question constitute the Data Availability Statement and **will be published in the article**, if accepted.<br><br>**Important:** Stating 'data available on request from the author' is not sufficient. If your data are only available upon request, select 'No' for the first question and explain your exceptional situation in the text box.<br><br>Do the authors confirm that all data underlying the findings described in their manuscript are fully available without restriction?	Yes - all data are fully available without restriction
**Describe where the data may be found in full sentences. If you are copying our sample text, replace any instances of XXX with the appropriate details.**<br><br>• If the data are **held or will be held in a public repository**, include URLs, accession numbers or DOIs. If this information will only be available after acceptance, indicate this by ticking the box below. For example: *All XXX files are available from the XXX database (accession number(s) XXX, XXX.*).<br>• If the data are all contained **within the manuscript and/or Supporting Information files**, enter the following: *All relevant data are within the manuscript and its Supporting Information files.*<br>• If neither of these applies but you are able to provide **details of access elsewhere**, with or without limitations, please do so. For example:	All the data is available in the manuscript.

Data cannot be shared publicly because of [XXX]. Data are available from the XXX Institutional Data Access / Ethics Committee (contact via XXX) for researchers who meet the criteria for access to confidential data.<br><br>*The data underlying the results presented in the study are available from (include the name of the third party and contact information or URL).*<br>• This text is appropriate if the data are owned by a third party and authors do not have permission to share the data.<br><br>* typeset	
Additional data availability information:	Tick here if the URLs/accession numbers/DOIs will be available only after acceptance of the manuscript for publication so that we can ensure their inclusion before publication.

Powered by Editorial Manager® and ProduXion Manager® from Aries Systems Corporation

Cover Letter

Click here to access/download;Manuscript;anxiety on medical and surgical plos amended.doc

Anxiety and associated factors among medical and surgical patients in Ethiopia: A systematic review and meta-analysis

Gidey Rtbey[1*], Milen Mihertabe[1], Fantahun Andualem[1], Mamaru Melkam[1], Setegn Fentahun[1]

Affiliations

[1] Department of Psychiatry, College of Medicine and Health Sciences, University of Gondar, Gondar, Ethiopia

Email address

GR: gidur2006@gmail.com

MM: milenmihertabe@gmail.com

FA: fantaandu27@gmail.com

MM: mamarumelkam@gmail.com

SF: setegnf21@gmail.com

*Corresponding author: Gidey Rtbey Email: gidur2006@gmail.com

Phone no. +251938146886/918140681

Abstract

Background: Anxiety, in addition to being a natural emotional response to any stressor, it can also have physical and psychological components. Patients diagnosed with chronic medical illness and patients appointed to undergo surgical procedures face various degrees of anxiety due to doubts related to the outcome of surgery, and the socioeconomic and psychological costs of the medical illness. This can have a negative impact on achieving the ultimate goals of managing the health problem and decreases their quality of life. In Ethiopia even though different studies were conducted on anxiety and associated factors both among medical and surgical patients, the findings were found to be inconsistent and had a wide discrepancy. So, this systematic review and meta-analysis revealed the estimated pooled effect size of anxiety among this population and guides to plan appropriate intervention at a national level.

Methods: Observational studies conducted on anxiety and associated factors among medical and surgical patients in Ethiopia were included based on the criteria after independent selection by two authors. Data were extracted using a Microsoft Excel spreadsheet and then exported to STATA version-11 for analysis. The random-effect model was used to estimate the pooled effect size of anxiety and its determinants from the selected studies with 95% confidence intervals. Funnel plots analysis and Egger regression tests were employed to check the presence of publication bias. Sub-group analysis and sensitivity analysis were also employed.

Result: A total of 8490 study participants were included from the twenty-three selected studies in this meta-analysis and systematic review. The pooled prevalence of anxiety among medical and surgical patients in Ethiopia was found to be 48.82% with 95 CI (42.66, 54.99). The subgroup analysis revealed that anxiety was highest in Oromia region 54.11%, 95% CI (40.69, 54.99), among surgical patients 56.97 %, 95% CI (49.79, 64.15), and studies that used the S-STAI tool 57.55%, 95% CI (51.30, 63.79). Being female [OR=2.84(2.02, 4.01)], fear of death [OR=2.93(1.57, 5.50)], and history of previous surgery[AOR=0.42(0.27, 0.065)] among surgical patients and being female [AOR= 2.35(1.94, 2.850] having poor social support [OR=2.22(1.62, 3.05)], perceived stigma [OR=4.25(1.97, 9.18)] and family history of mental illness [AOR=1.86(1.21, 2.86)] among medical patients were significantly associated with anxiety in this systematic review and meta-analysis.

Conclusion and recommendation: The pooled prevalence of anxiety in this systematic review and meta-analysis in Ethiopia was found to be high among medical and surgical patients. Therefore, it would be good for health care professionals to assess patients for anxiety in addition to managing their medical or surgical cases to early detection and intervene in them.

Keywords: Anxiety, medical patients, preoperative surgical patients, meta-analysis, Ethiopia.

Introduction

Anxiety is described as a feeling of emotional unease, distress, apprehension, or apprehensive concern that is subjective, accompanied by somatic and autonomic symptoms, and that could impairs individuals functioning (1). In addition to being a natural emotional response to risk, anxiety can also have physical and psychological components(1, 2).

Surgery is a potentially life-threatening treatment that makes the patient feel directly physically restrained. Patients who are having surgery may feel uneasiness, dread of not being able to awaken from anesthesia, fear of discomfort following the operation, and fear of dying(3). Irrespective of the type of surgery being performed, it is a procedure that could induce anxiety, which could cause patients to suffer varying degrees of worry(2, 4).

One of the most prevalent and incapacitating neuropsychiatric symptoms brought on by chronic medical illnesses like cancer, aside from surgical operations, is the appearance of anxiety symptoms(5). In the beginning, cancer patients feel shock or denial, followed by emotional turmoil, anxiety, lack of focus, difficulty falling asleep, loss of appetite, irritability, and intrusive worries about the future(6). As they cope with the effects of the diagnosis and deal with the challenges including decreased life expectancies, challenging therapy regimens, stigma, and loss of social and familial support, people with HIV/AIDS and other chronic medical illnesses frequently experience anxiety(7).

A meta-analysis revealed that the global pooled prevalence of preoperative anxiety among surgical patients was 48%(8) and 55.7% was found among surgical patients in low and middle income countries(9). In Ethiopia the prevalence of anxiety among surgical patients ranges between 47% and 70%(10, 11). On the other hand, manifestations of anxiety symptoms among patients with chronic medical illnesses like cancer, HIV/AIDS, hypertension and diabetes mellitus and kidney disease is common.

The consequences of anxiety among surgical patients acute myocardial infarction, heart failure, pulmonary edema, high readmission rate, poor quality of life and high rate of cardiac mortality which correlate with high postoperative pain, increased analgesic and anesthetic consumption, prolonged hospital stay, adverse influence during anesthetic induction and patient recovery and decrease patient satisfaction with perioperative care(12-15).

In individuals with chronic conditions, anxiety symptoms can have a variety of detrimental effects on their health than the general population(16). Untreated and unrecognized anxieties have negative repercussions on health, such as medication non-adherence, rapid disease progression, and poor health outcomes(17). Because anxiety can be so crippling, it can have a negative impact on a person's daily functioning and quality of life especially in case of diabetes mellitus it increases mortality rate(18). Besides this, comorbidity of this disorders have an

adverse economic impact on the patient, family, and the community at large due to the costs involved with diagnosis, treatment, and lost productivity(19).

Recent studies have shown that the type of surgery, the patient's gender, how they interact with the medical personnel, whether they have had surgery before, and how sensitive they are to stressful situations are all factors that contribute for anxiety in surgical patients(20-22). Furthermore, concern about the results of surgery (29.3%), worry about the recovery process (19.5%), and worry about complications (11.4%) were the most often reported significant causes of preoperative anxiety(23). While being widowed or divorced, being a woman, having a low level of education, being unemployed, having a high body mass index, having low income, lacking in social support, patients' residence, substance abuse, including alcohol, and non-adherence to medication were all linked to anxiety in patients with chronic medical conditions(18, 24-26).

Medical and surgical patients' health status is expected to be significantly improved as a result of the resources allocated to their health care within the country. Unluckily, there are some circumstances that compromise patients' outcome and prevent us from accomplishing these ultimate goals. To address such kind of hindrances, evidence about the burden of anxiety and its determinants is in demand at a national level. This study was aimed at determining the pooled effect size of anxiety and factors associated with it among chronically ill medical patients and surgical patients in Ethiopia. So, having such evidence-based data will be helpful to plan and carry out possible interventions at a country level.

Methods and materials

Protocol and registration

The protocol for this systematic review and meta-analysis was registered in the international prospective register of systemic review (PROSPERO) (ID=CRD42023427023).

Study design

Meta-analysis and systematic review were performed according to the Preferred Reporting Items for Systematic Review and Meta-Analysis (PRISMA-P 2020) standard(27)(**Supplementary Material 1**).

Searching strategies

A review all available research articles were done using the following databases: PubMed/MEDLINE, Scopus, African-wider, PsycINFO, EMBASE, Google Scholar, AJOL, World Health Organization (WHO) reports, and Cochrane library. In addition to this, a search of other gray literature was tried by searching Google, Google Scholar, and other internet search engines to search any additional articles until the end of May 2023. The search terms used were anxiety and associated factors among patients with chronic medical conditions and those undergoing surgery OR other Medical Subject Heading (Mesh), keywords, and free text search terms. The authors used several phrases, such as anxiety OR anxiety condition, medical and surgical patients, and combined terms by utilizing Boolean operators while searching for the literature that was already available. The terms that were in use to search the articles were ("anxiety OR anxiety disorder") AND ("associated factors" OR "determinants" AND "chronic medically ill patients" AND "surgical patients" OR "patients undergoing surgery" AND "Ethiopia").

Eligibility criteria

All peer-reviewed journal articles and grey literatures that addressed anxiety and associated factors among patients with chronic medical illness and patients undergoing surgery and written in English language were included in this meta-analysis and systematic review. All observational studies using various study designs on anxiety among medical and surgical patients in Ethiopia were included. Studies that lacks an abstract, don't have a full text, or were difficult to get the necessary data were excluded from this systematic review and meta-analysis.

Outcome variable measurement tool

The outcome variable of this systematic review and meta-analysis was anxiety in which it was assessed using different tools in the included studies. Among the included studies 11 of them were assessed using hospital anxiety depression scale (HADS), 7 were using state-trait anxiety

inventory scale (S-STAI) and the rest 5 were used other tools (PITI, HAS, GAD-7, DAS and BAIS). The 14-item HADS questionnaire is used to check for symptoms of anxiety and depression. Its internal consistency for anxiety was 0.78 and it was approved for local usage in Ethiopia. The scales use a cut-off point of $\geq$ 8 for anxiety(28). State-Trait Anxiety Inventory Scale (S-STAI), which is validated in Ethiopia(29). The State-Trait Anxiety Inventory Scale (S-STAI) features two subscales with a total of 40 items, 20 of which are assigned to the S-Anxiety and T-Anxiety subscales. The State Anxiety Scale (S-Anxiety), which measures subjective feelings of dread, tension, uneasiness, worry, and activation/arousal of the autonomic nervous system, assesses the current state of anxiety by asking respondents how they feel "right now." The Trait Anxiety Scale (T-Anxiety) measures relatively constant features of "anxiety proneness," such as overall feelings of calm, assurance, and security(30).

Data extraction

After carefully examining the papers' titles, abstracts, and full texts, the pertinent data were extracted using a Microsoft Excel Spreadsheet. All researches that have been endorsed by the reviewers were included. The data was extracted using the name of the principal investigator, years of publications, study design, study region, case type (medical or surgical), and sample size from the included articles. Additionally, the estimated combined effects of anxiety and its associated factors were retrieved along with their 95% confidence intervals. GR conducted the primary data extraction, and then FA assessed the extracted data independently. Disagreements and discrepancies were resolved through discussion with the third author MM (the fourth author) (**Supplementary material 3**).

Quality appraisal of the selected studies

To independently assess the quality of the research articles identified in this meta-analysis and systematic review, two authors (SF and MM (the second author)) used the standard instrument. The Joanna Briggs Institute (JBI) was used to assess the quality of the studies(31). This quality measuring instrument was developed for articles that reported prevalence data and observational studies. The assessment of the quality of the articles was the same because the study design was the same for all (cross-sectional). Articles with the final rating scale of five and above out of nine points were included in this meta-analysis and systematic review. Differences between reviewers

in quality ratings were resolved in a discussion with other third author (GR) to reach a common agreement (**Supplementary material 2**).

Data processing and analysis

The extracted data was exported to STATA version-11 statistical software for analysis. The estimated pooled effect size and the effect size of all articles with their 95% confidence intervals were obtained using a random-effects meta-analysis model. For the graphical summary, forest plot was used to estimate the pooled effect size and weight of each recruited study with 95% CI. The index of heterogeneity (I^2 statistics) was used to determine how heterogeneous the included articles were(32). After confirming the presence of heterogeneity; sub-group analysis employing the study region, case type (medical or surgical), and assessment tool used were done. Egger's weighted regression tests and Funnel plot analysis were also used to check publication bias(33, 34). After confirming the presence of publication bias in egger's test (0.039) trim and fill analysis was done to adjust the observed publication bias(35)(**Error! Reference source not found.**).

Res[illegible]

Study selection process

Using various search databases, 1990 studies were first identified, and the extra records led to the addition of 90 more studies. Due to duplication, 745 papers were removed, and 1190 studies were excluded because their titles were irrelevant or their goals were unrelated to this investigation. Out of the 145 studies that underwent full text evaluation, only 23 matched the qualifying requirements for this systematic review and meta-analysis (**Error! Reference source not found.**).

Characteristics of studies included

This meta-analysis and systematic review included a total of 23 studies on anxiety and associated factors among chronic medical and surgical patients in Ethiopia. Out of these nine articles were conducted in Amhara, six in Addis Ababa, four in Oromia, the final three in SNNPs, and one in the Somalia region (

Table 1).

The pooled prevalence of anxiety among medical and surgical patients

The pooled prevalence of anxiety among chronic medically ill and surgical patients in Ethiopia was found to be 48.82% with 95 CI (42.66, 54.99). In this meta-analysis and systematic review, the pooled prevalence of anxiety in Amhara, Addis Ababa, Oromia, SNNPs, and Somalia region was 48.7%, 49.7%, 54.11%, 47.14% and 29.8% respectively (**Error! Reference source not found.**).

Heterogeneity and publication bias

Heterogeneity was identified in this meta-analysis and systematic review with an I^2 of 97.3% and a P-value of 0.001. To determine whether the chosen articles might have a publication bias, a funnel plot was used. The absence of publication bias in the included papers is indicated by the funnel plot's placement inside the triangle, which is a symmetric distribution (**Error! Reference source not found.**). Egger's regression test was used to check publication bias and confirmed that there was a publication bias (P= 0.039) (Table 2). To adjust this publication bias trim and fill analysis was employed (**Error! Reference source not found.**).

Subgroup analysis

After it was detected that there was a heterogeneity, a subgroup analysis based on region, case type (medical and surgical) and assessment tools were employed. The Amhara region had a 48.7% of pooled level of anxiety among medical and surgical patients, 49.7% in Addis Ababa, 54.11% in Oromia and 42.59% in other regions. Analysis based the case type revealed that pooled prevalence was 56.97% among surgical and 43.63% medical patients whereas 57.55%, 44.66%, and 45.85% were pooled prevalence in studies which used S-STAI, HADS, and other assessment tools respectively (Table 3).

A leave-out-one sensitivity analysis

By omitting one study at a time, the sensitivity analysis was used to see how each study's findings affected the overall prevalence of anxiety in this meta-analysis and systematic review. The sensitivity analysis showed that all values were almost within the estimated 95% CI, indicating that the prevalence of this meta-analysis was not significantly affected by the removal of a single study (Table 4).

Associated factors with anxiety among medical and surgical patients

In this meta-analysis and systematic review, factors contributing for anxiety among surgical patients in Ethiopia were identified. Being female sex was found to be 2.84 times more likely to have anxiety than males in this systematic review and meta-analysis [OR=2.84(2.02, 4.01)].

Having fear of death among surgical patients was also a contributing factor for anxiety in this study. Participants who had fear of death were 2.93 times higher in developing anxiety compared to their counterparts [OR=2.93(1.57, 5.50)].

The other variable associated with anxiety was having previous history of surgery. Surgical patients who had previous history of surgery were 58% less likely to have preoperative anxiety than participants who didn't have previous history of surgery (**Error! Reference source not found.**).

Regarding to variables associated with anxiety among chronic medical patients in this systematic review and meat-analysis, being female was higher in developing anxiety than males [AOR=2.35(1.94, 2.85)].

The other predictive variable which significantly associated with anxiety among medical patients was having poor social support. In this review, medical patients with poor social support were 2.22 at risk of developing anxiety compared to those who had strong social support [AOR=2.22(1.62, 3.05)].

Patients who had perceived stigma were more than four times higher to have anxiety than their counterparts [OR=4.25(1.97, 9.18)]. Having family history mental illness was also associated with anxiety among medical patients [AOR=1.86(1.21, 2.86)] (**Error! Reference source not found.**).

Discussion

This systematic review and meta-analysis synthesized the findings of 23 primary investigations carried out among medical and surgical patients in Ethiopia, to ascertain the pooled prevalence and factors associated with anxiety among 8490 study participants.

The pooled prevalence of anxiety among medical and surgical patients in Ethiopia was found to be 48.82% with 95 CI (42.66, 54.99). This result was consistent with a global meta-analysis among patients undergoing surgery (48%)(8) and studies conducted in Nigeria (51%)(57) Rwanda (52.1%)(58), Vietnam (43%)(59), Pakistan(45.95%)(60) and Turkey (52.2%)(61). The pooled estimate of this review was higher compared to studies conducted in Kenya (37%)(62) China (15%)(63), India (31%)(64) and Brazil (35%)(65). The possible explanation for this discrepancy could be due to sociocultural and socioeconomic differences. Moreover, access to information regarding their surgical procedure or medical illness, variations in health care delivery system and tools used to screen anxiety might contributed for this inconsistency.

However, the pooled estimate of this review was lower than studies carried out in Tunisia 67.5%(66), Nepal (70.6%) (67) and USA (72.7%) (20). This variation could be due to the assessment tool differences and type of case in which the participants had. Studies conducted

among patients undergoing surgical procedure that could have greater impact in increment of anxiety among this population may partly explain this variation. Furthermore, study participants' variations for instance; the study conducted in USA recruited only females whom they are more likely to experience anxiety than males.

In the subgroup analysis, the pooled prevalence anxiety in Oromia region was higher in which this could be due tool and case type differences. Furthermore, prevalence of anxiety among studies assessed using S-STAI was higher compared to other tools. This could be as a result of psychometric properties of the tool and the tool was also used among patients undergoing surgery in which could increase the pooled effect size of the anxiety.

When compared to medical patients, surgical patients were found to have a higher pooled prevalence of anxiety. This could be explained by the possibility that anxiety in those who were about to have surgery was likely to be high due to an emotional and physiological reaction to the anticipated concern. Additionally, S-STAI was utilized in the majority of studies including surgical cases, which might have indirectly influenced the results.

Regarding to predictive variables to anxiety, in this systematic review and meta-analysis female patients were more than two times higher in developing anxiety than males among both medical and surgical patients which was supported by studies conducted in Brazil(24), India(68) and China(69). The possible reason for this might be women's affective responses to stressors, domestic violence, learned helplessness, hormonal changes associated with menstruation, lower income, and lower social status(70). In addition, the existence of specific mental health conditions in women, such as premenstrual dysphoric disorder, postpartum depression, and postmenopausal mental health conditions, which are linked to hormonal changes, may raise the possibility that female patients feeling anxious(71).

Surgical patients who had fear of death during undergoing surgery were around three times more likely to develop anxiety compared to their counterparts in this systematic review and meta-analysis. This evidence was consistent with survey done in USA to assess fear of death and anesthesia complication in patients undergoing surgery(72). This might be due to the anesthesia medication complications or the surgical procedure by itself patients perceive it would be unimaginable to wake up after the procedure is done (73).

This systematic review and meta-analysis also revealed that having poor social support was associated with anxiety among medical patients. This result was supported by studies from Nepal (74), USA(75) and Pakistan (76). This could be possibly due to the effect of getting insufficient emotional, instrumental and informational support from significant other which leads to cope poorly in stressful situations and decrease their resilience(77). Participants with chronic medical illness who had perceived stigma were more than four times more likely to have anxiety than those who didn't have perceived stigma in which this was consistent with studies from Malawi

(78) and China(79). This could be as result of stigmatized persons may internalize perceived prejudices and develop negative feelings about themselves and patients feel shame and embarrassment about having the debilitating illness and bother about their social life which could prone them for developing anxiety(80).

Having had family history mental illness was also an independent variable which found to be associated with anxiety among medical patients in this review. This finding was supported by study conducted in Nepal(74). Family history of mental illnesses is one risk factor for mental disorders (the hereditary effect), notably mood disorders, which carries a risk of between 10 and 25 percent for a child if one parent has a mood disorder(81).

Limitations of the study

Although this meta-analysis has a great advantage, to give the pooled effect of anxiety among medical and surgical patients in Ethiopia but it has limitations. The studies included in this review were all cross-sectional studies in which this finding couldn't show the temporal relationship of anxiety and its determinants.

Conclusion and recommendation

The pooled prevalence of anxiety among medical and surgical patients in Ethiopia was found to be 48.82%. Fear of death and history of surgery among surgical patients, poor social support, having perceived stigma, and family history of mental illness among medical patients were associated with anxiety whereas being female was associated with outcome variable among both cases. Therefore, health professional should routinely assess patients for anxiety besides treating medical and surgical illnesses to achieve the ultimate goal of patients care and to impact positively on the prognosis these conditions.

Declaration

Ethics approval and consent to participate

Not applicable

Consent for publication

Not applicable

Availability of data and materials

All the data is present in the manuscript.

Competing interests

The authors declared that they have no competing interests.

Funding

Funding is not applicable

Authors' contributions

GR conceptualized the study, involved in design, analysis, interpretation, and manuscript writing. MM, GN, and SF made a substantial contribution to the extract data, analysis, and FA and MM took part in the interpretation, drafting of the manuscript, and critical revision of important intellectual content. All the authors read and approved the final manuscript.

Acknowledgments

Our gratitude goes to the authors who conducted the primary investigations which were included in this systematic review and meta-analysis.

Abbreviation/acronyms

AIDS: Acquired Immune deficiency Syndrome, AOR: Adjusted Odd Ratio, CI: Confidence Interval, BAIS: Beck Anxiety Inventory Scale, GAD-7: Generalized Anxiety Disorder 7-item, HADS: Hospital Anxiety and Depression Scale, HAS: Hospital Anxiety Scale, DASS-21: Depression Anxiety and Stress Scale, PITI: Preoperative Intrusive Thought Inventory, PRISMA: Preferred Reporting Items for Systematic Review and Meta-Analysis, SNNPs: South Nation Nationality and Peoples, S-STAI: State-Trait Anxiety Inventory Scale, USA: United States of America, and WHO: World Health Organization.

References

1. Edition F. Diagnostic and statistical manual of mental disorders. Am Psychiatric Assoc. 2013;21(21):591-643.
2. Bellani ML. Psychological aspects in day-case surgery. International journal of surgery. 2008;6:S44-S6.
3. Akıncı S, Sarıcaoglu F, Dal D. Preoperative anesthesic evaluation. Hacettepe Med J. 2003;36:91-7.
4. Bailey L. Strategies for decreasing patient anxiety in the perioperative setting. AORN journal. 2010;92(4):445-60.
5. Carroll BT, Kathol RG, Noyes Jr R, Wald TG, Clamon GH. Screening for depression and anxiety in cancer patients using the Hospital Anxiety and Depression Scale. General hospital psychiatry. 1993;15(2):69-74.
6. Trill MD. Anxiety and sleep disorders in cancer patients. EJC supplements. 2013;11(2):216.
7. Owe-Larsson B, Sall L, Salamon E, Allgulander C. HIV infection and psychiatric illness. African journal of psychiatry. 2009;12(2):115-28.
8. Abate SM, Chekol YA, Basu B. Global prevalence and determinants of preoperative anxiety among surgical patients: A systematic review and meta-analysis. International Journal of Surgery Open. 2020;25:6-16.
9. Bedaso A, Mekonnen N, Duko B. Prevalence and factors associated with preoperative anxiety among patients undergoing surgery in low-income and middle-income countries: a systematic review and meta-analysis. BMJ open. 2022;12(3):e058187.
10. Bedaso A, Ayalew M. Preoperative anxiety among adult patients undergoing elective surgery: a prospective survey at a general hospital in Ethiopia. Patient safety in surgery. 2019;13(1):1-8.
11. Afrassa N, Kassa RN, Legesse TG. Preoperative anxiety and its associated factors among patients undergoing cardiac catheterization at saint peter Specialized Hospital and Addis Cardiac Center, Addis Ababa, Ethiopia. International Journal of Africa Nursing Sciences. 2022;17:100430.

12. Stamenkovic DM, Rancic NK, Latas MB, Neskovic V, Rondovic GM, Wu JD, et al. Preoperative anxiety and implications on postoperative recovery: what can we do to change our history. Minerva anestesiologica. 2018;84(11):1307-17.
13. Chow CH, Van Lieshout RJ, Schmidt LA, Dobson KG, Buckley N. Systematic review: audiovisual interventions for reducing preoperative anxiety in children undergoing elective surgery. Journal of pediatric psychology. 2016;41(2):182-203.
14. Cserép Z, Losoncz E, Balog P, Szili-Török T, Husz A, Juhász B, et al. The impact of preoperative anxiety and education level on long-term mortality after cardiac surgery. Journal of Cardiothoracic Surgery. 2012;7:1-8.
15. Joseph HK, Whitcomb J, Taylor W. Effect of anxiety on individuals and caregivers after coronary artery bypass grafting surgery: a review of the literature. Dimensions of Critical Care Nursing. 2015;34(5):285-8.
16. Carrico AW, Bangsberg DR, Weiser SD, Chartier M, Dilworth SE, Riley ED. Psychiatric correlates of HAART utilization and viral load among HIV-positive impoverished persons. AIDS (London, England). 2011;25(8):1113.
17. Li H, Ge S, Greene B, Dunbar-Jacob J. Depression in the context of chronic diseases in the United States and China. International journal of nursing sciences. 2019;6(1):117-22.
18. Naicker K, Johnson JA, Skogen JC, Manuel D, Øverland S, Sivertsen B, et al. Type 2 diabetes and comorbid symptoms of depression and anxiety: longitudinal associations with mortality risk. Diabetes care. 2017;40(3):352-8.
19. Mausbach BT, Yeung P, Bos T, Irwin SA. Health care costs of depression in patients diagnosed with cancer. Psycho- oncology. 2018;27(7):1735-41.
20. Maheshwari D, Ismail S. Preoperative anxiety in patients selecting either general or regional anesthesia for elective cesarean section. Journal of anaesthesiology, clinical pharmacology. 2015;31(2):196.
21. Robertson A, Khan R, Fick D, Robertson WB, Gunaratne DR, Yapa S, et al., editors. The effect of Virtual Reality in reducing preoperative anxiety in patients prior to arthroscopic knee surgery: A randomised controlled trial. 2017 IEEE 5th International Conference on Serious Games and Applications for Health (SeGAH); 2017: IEEE.
22. Duggan M, Dowd N, O'Mara D, Harmon D, Tormey W, Cunningham AJ. Benzodiazepine premedication may attenuate the stress response in daycase anesthesia: a pilot study. Age (yr). 2002;34(11.2):38.4-11.9.
23. Kuzminskaitė V, Kaklauskaitė J, Petkevičiūtė J. Incidence and features of preoperative anxiety in patients undergoing elective non-cardiac surgery. Acta Medica Lituanica. 2019;26(1):93.
24. Campos LN, Guimarães MDC, Remien RH. Anxiety and depression symptoms as risk factors for non-adherence to antiretroviral therapy in Brazil. AIDS and Behavior. 2010;14:289-99.
25. Edmealem A, Olis CS. Factors associated with anxiety and depression among diabetes, hypertension, and heart failure patients at Dessie Referral Hospital, Northeast Ethiopia. Behavioural neurology. 2020;2020.
26. Nawaz MS, Shah KU, Rashid HU, Mahmood S, Bukhsh A, Rehman IU, et al. Factors associated with anxiety in type 2 diabetes mellitus patients in Pakistan. International Journal of Diabetes in Developing Countries. 2018;38:298-304.
27. Page MJ, McKenzie JE, Bossuyt PM, Boutron I, Hoffmann TC, Mulrow CD, et al. The PRISMA 2020 statement: an updated guideline for reporting systematic reviews. International journal of surgery. 2021;88:105906.
28. Reda AA. Reliability and validity of the Ethiopian version of the hospital anxiety and depression scale (HADS) in HIV infected patients. PLoS One. 2011;6(1):e16049.
29. Mulatu MS. Psychometric properties of scores on the preliminary Amharic version of the State-Trait Anxiety Inventory in Ethiopia. Educational and Psychological Measurement. 2002;62(1):130-46.

30. Spielberger C. Manual for the State-Trait Anxiety Inventory STAI (form Y)("self-evaluation questionnaire"). Menlo Park. CA: Mind Garden, Inc location. 1983.
31. Munn Z, Moola S, Lisy K, Riitano D, Tufanaru C. Methodological guidance for systematic reviews of observational epidemiological studies reporting prevalence and cumulative incidence data. JBI Evidence Implementation. 2015;13(3):147-53.
32. Higgins JP, Thompson SG. Quantifying heterogeneity in a meta- analysis. Statistics in medicine. 2002;21(11):1539-58.
33. Egger M, Smith GD, Schneider M, Minder C. Bias in meta-analysis detected by a simple, graphical test. Bmj. 1997;315(7109):629-34.
34. Sterne JA, Egger M. Funnel plots for detecting bias in meta-analysis: guidelines on choice of axis. Journal of clinical epidemiology. 2001;54(10):1046-55.
35. Duval S, Tweedie R. Trim and fill: a simple funnel- plot–based method of testing and adjusting for publication bias in meta- analysis. Biometrics. 2000;56(2):455-63.
36. Ferede YA, Bizuneh YB, Workie MM, Admass BA. "Prevalence and associated factors of preoperative anxiety among obstetric patients who underwent cesarean section": A cross-sectional study. Annals of Medicine and Surgery. 2022;74:103272.
37. Mulugeta H, Ayana M, Sintayehu M, Dessie G, Zewdu T. Preoperative anxiety and associated factors among adult surgical patients in Debre Markos and Felege Hiwot referral hospitals, Northwest Ethiopia. BMC anesthesiology. 2018;18(1):1-9.
38. Nigussie S, Belachew T, Wolancho W. Predictors of preoperative anxiety among surgical patients in Jimma University Specialized Teaching Hospital, South Western Ethiopia. BMC Surgery. 2014;14(1):67.
39. Shewangzaw Engda A, Belay Yigzaw H, Alemnew Engdaw N, Admasu Basha E, Adem A, Dargie Wubetu A, et al. Magnitude of Preoperative Anxiety and Associated Factors Among Adult Surgical Patients in Debre Berhan Comprehensive Specialized Hospital. International Journal of General Medicine. 2022:5999-6007.
40. Srahbzu M, Yigizaw N, Fanta T, Assefa D, Tirfeneh E. Prevalence of depression and anxiety and associated factors among patients visiting orthopedic outpatient clinic at Tikur Anbessa specialized Hospital, Addis Ababa, Ethiopia, 2017. J Psychiatry. 2018;21(2):10.4172.
41. Woldegerima Y, Fitwi G, Yimer H, Hailekiros A. Prevalence and factors associated with preoperative anxiety among elective surgical patients at University of Gondar Hospital. Gondar, Northwest Ethiopia, 2017. A cross-sectional study. International Journal of Surgery Open. 2018;10:21-9.
42. Wondmieneh A. Preoperative anxiety and associated factors among adult elective surgery patients in North Wollo Zone, Northeast Ethiopia. Open Access Surgery. 2020:85-94.
43. Aberha M, Gebeyehu A, Ayano G. Prevalence and factors associated with anxiety among patients with hypertension on follow up at Menelik-II Referral Hospital, Addis Ababa Ethiopia. J Psychiatry. 2016;19(4):378.
44. Atinafu BT, Demlew TM, Tarekegn FN. Magnitude of anxiety and depression and associated factors among palliative care patients with cancer at Tikur Anbessa specialized Hospital, Ethiopia. Ethiopian Journal of Health Sciences. 2022;32(2).
45. Ayalew M, Deribe B, Duko B, Geleta D, Bogale N, Gemechu L, et al. Prevalence of depression and anxiety symptoms and their determinant factors among patients with cancer in southern Ethiopia: a cross-sectional study. BMJ open. 2022;12(1):e051317.
46. Belete A, Andaregie G, Tareke M, Birhan T, Azale T. Prevalence of anxiety and associated factors among people living HIV/AIDS at debretabor general hospital anti retro viral clinic Debretabor, Amhara, Ethiopia, 2014. Am J Psychiatry Neurosci. 2014;2(6):109-14.
47. Duko B, Toma A, Asnake S, Abraham Y. Depression, anxiety and their correlates among patients with HIV in South Ethiopia: An institution-based cross-sectional study. Frontiers in psychiatry. 2019;10:290.

48. Edmealem A, Olis CS. Factors Associated with Anxiety and Depression among Diabetes, Hypertension, and Heart Failure Patients at Dessie Referral Hospital, Northeast Ethiopia. Behavioural Neurology. 2020;2020:3609873.
49. Endeshaw D, Walle TA, Yohannes S. Depression, anxiety and their associated factors among patients with cancer receiving treatment at oncology units in Amhara Region, Ethiopia: a cross-sectional study. BMJ open. 2022;12(11):e063965.
50. Hajure M, Tariku M, Mohammedhussein M, Dule A. Depression, anxiety and associated factors among chronic medical patients amid COVID-19 pandemic in Mettu Karl Referral Hospital, Mettu, Ethiopia, 2020. Neuropsychiatric disease and treatment. 2020:2511-8.
51. Nigussie K, Sertsu A, Ayana GM, Dessie Y, Bete T, Abdisa L, et al. Determinants of depression and anxiety among type 2 diabetes patients in governments' hospitals at Harari regional state, Eastern Ethiopia: A multi-center cross-sectional study. BMC Psychiatry. 2023;23(1):13.
52. Tesfaw G, Ayano G, Awoke T, Assefa D, Birhanu Z, Miheretie G, et al. Prevalence and correlates of depression and anxiety among patients with HIV on-follow up at Alert Hospital, Addis Ababa, Ethiopia. BMC Psychiatry. 2016;16(1):368.
53. Tesfaw G, Siyoum M, Salelew E, Minichil W. Anxiety and the associated factors among admitted surgical and medical patients, Addis Ababa, Ethiopia: A cross-sectional study. 2022.
54. Tibebu NS, Kassie BA, Anteneh TA, Rade BK. Depression, anxiety and stress among HIV-positive pregnant women in Ethiopia during the COVID-19 pandemic. Transactions of The Royal Society of Tropical Medicine and Hygiene. 2023;117(5):317-25.
55. Tiki T. Prevalence and Associated Factors of Anxiety among Type 2 Diabetes Mellitus Patients on Follow Up at Ambo General Hospital, Oromia Regional State, Ethiopia, Institutional Based Cross Sectional Study. J Psychiatry Depress Anxiety. 2017;3(007).
56. Yousuf A, Musa R, Isa MLM, Arifin SRM. Anxiety and depression among women living with HIV: prevalence and correlations. Clinical practice and epidemiology in mental health: CP & EMH. 2020;16:59.
57. Akinsulore A, Owojuyigbe AM, Faponle AF, Fatoye FO. Assessment of preoperative and postoperative anxiety among elective major surgery patients in a tertiary hospital in Nigeria. Middle East J Anaesthesiol. 2015;23(2):235-40.
58. Uwayezu MG, Gishoma D, Sego R, Mukeshimana M, Collins A. Anxiety and depression among cancer patients: prevalence and associated factors at a Rwandan referral hospital. Rwanda Journal of Medicine and Health Sciences. 2019;2(2):118-25.
59. Truong DV, Bui QTT, Nguyen DT, Moore J. Anxiety among inpatients with cancer: findings from a hospital-based cross-sectional study in Vietnam. Cancer Control. 2019;26(1):1073274819864641.
60. Latif A, Khan RMS, Nawaz K. Depression and anxiety in patients undergoing elective and emergency surgery: Cross-sectional study from Allama Iqbal Memorial Teaching Hospital, Sialkot. Sialkot morbidity and mortality. 2017;8:9.
61. Dogu O, Aydemir Y. Anxiety and depression as emotional problems in patients with chronic heart, kidney and respiratory disorders. International Journal of Caring Sciences. 2018;11(1):543-9.
62. Chanda C. Assessment of preoperative anxiety amongst patients presenting for elective surgery at the Kenyatta National Hospital: University of Nairobi, Kenya; 2012.
63. Huang M, Liu Y, Wang J, Mo L, Wang Y, Chen L, et al. High rates of depression anxiety and suicidal ideation among inpatients in general hospital in China. International journal of psychiatry in clinical practice. 2019;23(2):99-105.
64. Vadhanan P, Tripaty DK, Balakrishnan K. Pre-operative anxiety amongst patients in a tertiary care hospital in India-a prevalence study. Journal of Society of Anesthesiologists of Nepal. 2017;4(1):5-10.
65. de Moraes VY, Jorge MR, Faloppa F, Belloti JC. Anxiety and Depression in Brazilian Orthopaedics Inpatients: A Cross Sectional Study with a Clinical Sample Comparison. Journal of Clinical Psychology in Medical Settings. 2010;17(1):31-7.

66. Zammit N, Menel M, Rania F. Preoperative anxiety in the tertiary care hospitals of Sousse, Tunisia: prevalence and predictors. SOJ Surg. 2018;5(1):1-5.
67. Ghimire R, Poudel P. Preoperative anxiety and its determinants among patients scheduled for major surgery: a hospital based study. J Anesthesiol. 2018;6(2):57-60.
68. Reuters T. Depression and anxiety among HIV-positive sex workers in Kolkata. India: testing and modifying the hospital anxiety depression scale. 2013.
69. Liu L, Pang R, Sun W, Wu M, Qu P, Lu C, et al. Functional social support, psychological capital, and depressive and anxiety symptoms among people living with HIV/AIDS employed full-time. BMC psychiatry. 2013;13(1):1-10.
70. Kanchan T, Menezes RG. Suicidal hanging in Manipal, South India–victim profile and gender differences. Journal of forensic and legal medicine. 2008;15(8):493-6.
71. Albert PR. Why is depression more prevalent in women? Journal of psychiatry & neuroscience: JPN. 2015;40(4):219.
72. Burkle C, Mann C, Steege J, Stokke J, Jacob A, Pasternak J. Patient fear of anesthesia complications according to surgical type: potential impact on informed consent for anesthesia. Acta Anaesthesiologica Scandinavica. 2014;58(10):1249-57.
73. Akutay S, Ceyhan Ö. The relationship between fear of surgery and affecting factors in surgical patients. 2023;12(1):22.
74. Sharma K, Dhungana G, Adhikari S, Bista Pandey A, Sharma M. Depression and anxiety among patients with type II diabetes mellitus in Chitwan Medical College Teaching Hospital, Nepal. Nursing research and practice. 2021;2021.
75. Barnes RT. Psychological distress in patients with orthopaedic trauma injuries: University of Florida; 2013.
76. Husain N, Humail SM, Chaudhry IB, Rahman R, Robinson H, Creed F. Psychological distress among patients of an orthopaedic outpatient clinic: a study from a low-income country. Annals of General Psychiatry. 2010;9(1):9.
77. Singer JE, Lord D. The role of social support in coping with chronic or life-threatening illness. Handbook of Psychology and Health (Volume IV): Routledge; 2020. p. 269-77.
78. Kamen C, Arganbright J, Kienitz E, Weller M, Khaylis A, Shenkman T, et al. HIV-related stigma: implications for symptoms of anxiety and depression among Malawian women. African Journal of AIDS Research. 2015;14(1):67-73.
79. Mo PKH, Lau JTF, Yu X, Gu J. A Model of Associative Stigma on Depression and Anxiety Among Children of HIV-Infected Parents in China. AIDS and Behavior. 2015;19(1):50-9.
80. Tucker JR, Hammer JH, Vogel DL, Bitman RL, Wade NG, Maier EJ. Disentangling self-stigma: Are mental illness and help-seeking self-stigmas different? Journal of counseling psychology. 2013;60(4):520.
81. Sadock BJ. Kaplan & Sadock's synopsis of psychiatry: behavioral sciences/clinical psychiatry: Wolters Kluwer Philadelphia, PA; 2015.

Table 1: Characteristics of studies selected in this systematic review and meta-analysis on anxiety and associated factors among medical and surgical in Ethiopia.

First author's name	Year of publication	Region	Sample size	Prevalence of anxiety (%)
N. Afrassa et al. (11).	2022	Addis Ababa	267	70.4
Bedaso and Ayalew(10).	2019	SNNPs	407	47
Y.A. Ferede et al.(36)	2022	Amhara	374	63
Mulugeta et al. (37)	2019	Amhara	353	61
Nigussie et al. (38)	2014	Oromia	239	70.3
Shewangzaw Engda et al. (39)	2022	Amhara	354	53.6
Srahbzu et al.(40)	2018	Addis Ababa	407	39.8
Woldegerima, YB et al.(41)	2018	Amhara	181	59.6
Wondmieneh and adam(42)	2020	Amahara	216	48.3
Aberha M et al(43)	2016	Addis Ababa	417	28.5
Bantalem T.A. et al. (44)	2022	Addis Ababa	171	64.9

Ayalew M, et al.(45)	2022	SNNPs	415	60
Amsalu Belete et al.(46)	2014	Amhara	443	22.2
Duko et al.(47)	2019	SNNPs	363	34.4
Edmealem and Sanchez (48)	2020	Amhara	404	32
Endeshaw D, et al.(49)	2022	Amhara	423	57.1
Hajure et al.(50)	2020	Oromia	423	61.8
Nigussie et al.(51)	2023	Oromia	421	40.4
Tesfaw et al.(52)	2016	Addis Ababa	417	32.4
Tesfaw et al.(53)	2022	Addis Ababa	590	62.7
N. S. Tibebu et al.(54)	2023	Amhara	423	42.1
Tiki T et al. (55)	2017	Oromia	423	44.2
Yousuf, A et al.(56)	2020	Somalia	357	28.9

Table 2: Egger's tests of anxiety among medical and surgical patients in Ethiopia.

**Std_Eff**	**Coef.**	**Std. Err.**	**t**	**P>t**	**[95% Conf.**	**Interval]**
slope	3.332214	19.97388	0.17	0.869	-38.20575	44.87018
bias	17.74579	8.047242	2.21	0.039	1.0106638	34.48095

Table 3: Subgroup analysis of anxiety and associated factors among medical and surgical patients in Ethiopia.

**Variables**	**Subgroup**	**Number of studies**	**Sample size**	**Prevalence (95% CI)**	**I^2 (%)**	**P-value**
**Region**	Amhara	9	3173	48.70(38.34, 59.03	97.4	0.001
	Addis Ababa	6	2686	49.70(35.12, 64.29)	98.2	$\leq$ 0.001
	Oromia	4	1503	54.11(40.69, 54.99)	96.6	0.001

	*Others	4	1542	42.59(28.83, 56.32)	97	0.001
**Case type**	Medical	14	5685	43.63(35.79, 51.47)	97.5	0.001
	Surgical	9	2800	56.97(49.79, 64.15)	93.7	0.001
**Assessment tool**	HADS	11	4237	44.68(36.79, 52.57)	96.6	0.001
	S-STAI	7	2126	57.55(51.30, 63.79)	88.7	0.001
	**Others	5	2127	45.85(27.96, 63.74)	98.8	0.001

*Others: SNNPs and Somalia. **Others: PITI, GAD-7, BAIS, HAS, DAS.

Table 4: Sensitivity analysis of anxiety and associated factors among medical and surgical in Ethiopia.

**Omitted studies**	**Estimated 95% CI**	**Heterogeneity**	
		**I^2 (%)**	**P-value**
N. Afrassa et al.	47.84(41.72, 53.97)	97.1	0.001
Bedaso and Ayalew	48.91(42.46, 55.37)	97.4	0.001
Y.A. Ferede et al	48.18(41.90, 54.45)	97.3	0.001
Mulugeta et al.	48.27(41.95, 54.59)	97.3	0.001
Nigussie et al.	47.85(41.71, 54.00)	97.2	0.001
Shewangzaw Engda et al.	48.61(42.2, 55.02)	97.4	0.001
Srahbzu et al.	49.24(42.81.55.66)	97.4	0.001

Woldegerima, YB et al	48.35(42.03, 54.67)	97.4	0.001
Wondmieneh and adam	48.85(42.47,55.23)	97.4	0.001
Aberha M et al.	49.75(43.56, 55.94)	97.1	0.001
Bantalem T.A. et al.	48.11(41.84,54.39)	97.3	0.001
Ayalew M, et al.	48.31(41.98, 54.65)	97.3	0.001
Amsalu Belete et al.	58.04(44.22, 55.86)	96.7	0.001
Duko et al.	49.48(43.14, 55.83)	97.3	0.001
Edmealem and Sanchez Olis	49.59(43.30, 55.89)	97.3	0.001
Endeshaw D, et al.	48.45(42.06,54.84)	97.3	0.001
Hajure et al.	48.23(41.93, 54.53)	97.3	0.001
Nigussie et al.	49.21(42.78, 55.65)	97.3	0.001
Tesfaw et al. 2016	49.58(43.27, 55.88)	97.3	0.001
Tesfaw et al. 2022	48.18(41.93, 54.44)	97.2	0.001
N. S. Tibebu et al.	49.13(42.69, 55.58)	97.4	0.001
Tiki T et al.	49.04(42.58, 55.50)	97.4	0.001
Yousuf, A et al.	49.73(43.51, 55.96)	97.2	0.001

Supplementary materials

1. PRISMA-2020 checklist for anxiety
2. Quality assessment scores for included studies
3. Extracted data for anxiety

Figure
Click here to access/download;Figure;Figure.pdf

Lorem Ipsum

Lorem ipsum dolor sit amet, consectetur adipiscing elit. Mauris maximus fringilla lig[illegible] in malesuada erat tempor ac. Quisque dapibus posuere turpis, vel aliquam massa vehicula non.

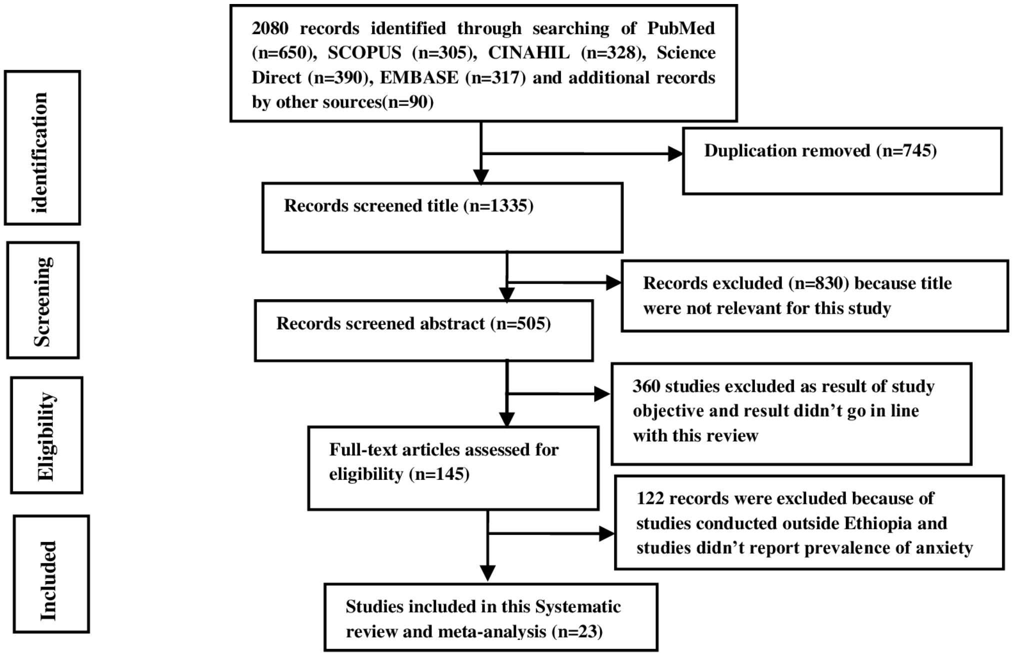

Figure 1. Flow chart describing the selection of studies conducted on anxiety among medical and surgical patients in Ethiopia.

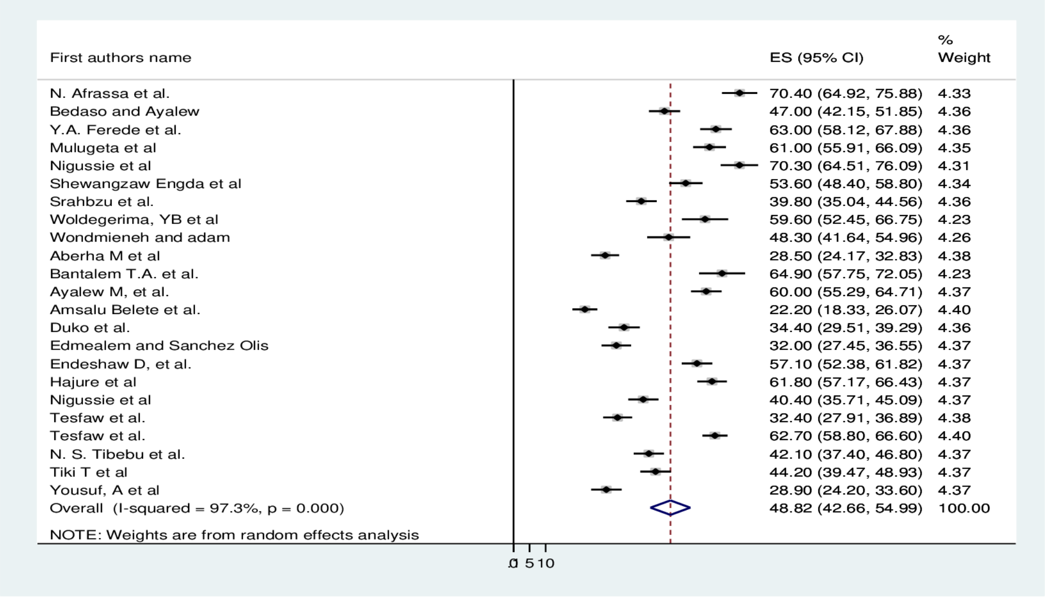

Figure 2. A Forest plot that shows the pooled level of anxiety among medical and surgical patients in Ethiopia.

test-test-1 This is a preview of your figure rendered on a simulated PLOS journal page.

Maecenas ac est sit amet odio sollicitudin euismod. In risus odio, convallis a neque ac, varius ultricies arcu. Vestibulum et quam iaculis, ultricies odio et, molestie magna. Suspendisse vehicula purus id turpis eleifend, et convallis dui dignissim. Praesent tempus elit a metus sollicitudin, sed fringilla nulla porttitor. Nullam in tempus massa. Nunc maximus magna massa, nec volutpat risus rhoncus ut. Fusce quis ante sem. Aenean nulla nibh, tempus sit amet rhoncus at, eleifend vel risus. Sed dictum, sem ultrices elementu[illegible] pharetra, lacus diam volutpat orci, scelerisque semper dui lacus ut enim.

Suspendisse in nunc id lacus commodo consequat. Proin semper aliquam varius. Fusce vitae neque aliquam nisi ultrices sodales vitae ut enim. Vivamus nec dictum ipsum. Sed condimentum ante eu urna tincidunt tincidunt. In ac lacus nec ipsum viverra volutpat posuere vel lacus. Class aptent taciti sociosqu ad litora torquent per conubia nostra, per inceptos himenaeos. Morbi rhoncus ipsum quis lorem hendrerit, at vulputate massa tempus. Ut arcu nisl, gravida vitae risus ultricies, porta venenatis massa. Cras dignissim, enim at faucibus aliquam, sapien nisl eleifend dolor, vel mollis nulla nisi id ipsum. Pellentesque vehicula ultricies risus sit amet faucibus. Praesent sit amet mi ac est faucibus accumsan. Praesent pulvinar sit amet orci auctor feugiat.

Lorem Ipsum

Lorem ipsum dolor sit amet, consectetur adipiscing elit. Mauris maximus fringilla ligula, in mal[illegible]da erat tempor ac. Quisque dapibus posuere turpis, vel aliquam massa vehicula non.

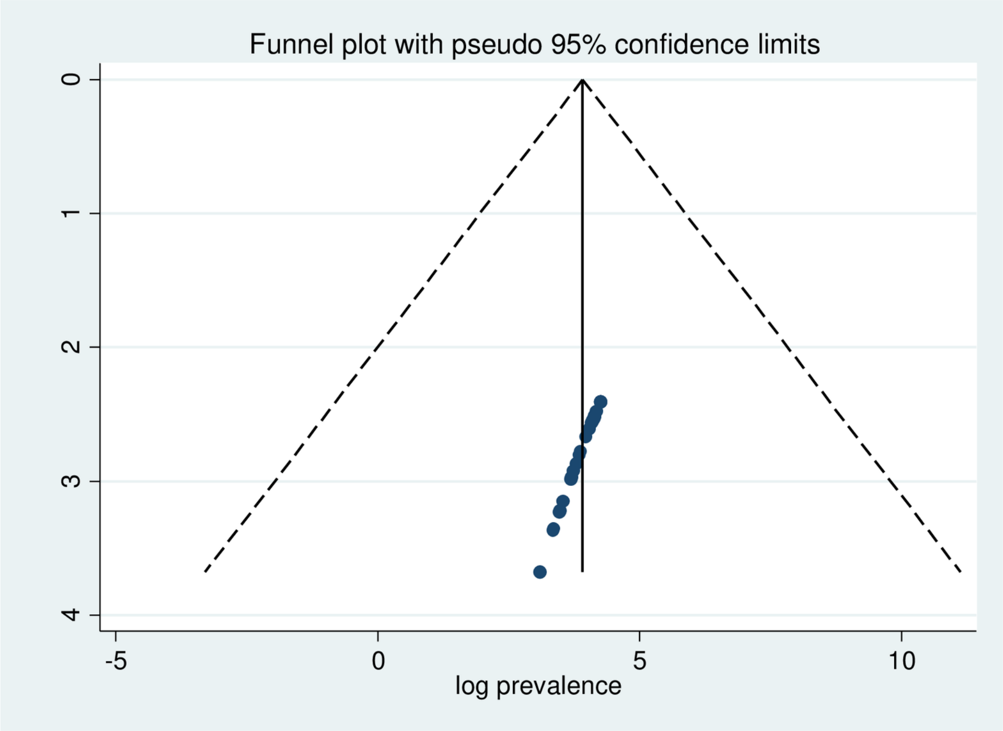

Figure 3. Funnel plot of anxiety among medical and patients in Ethiopia

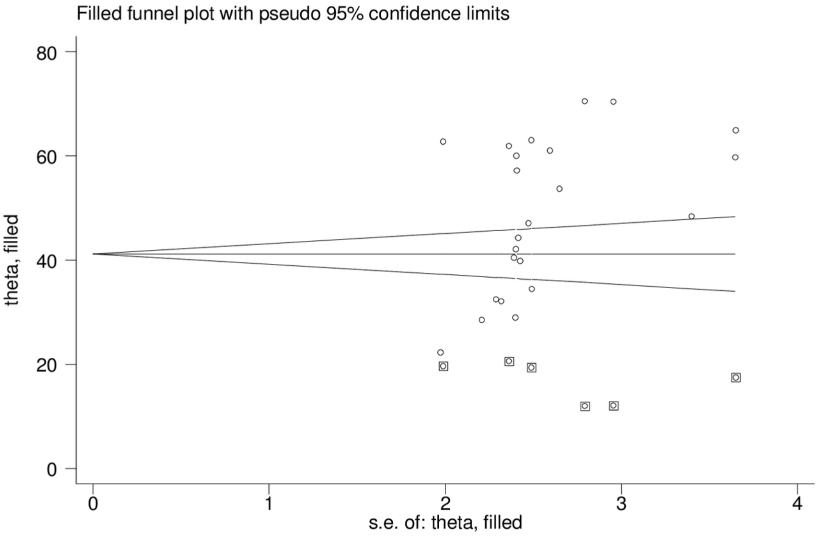

Figure 4. Trim and fill analysis for meta-analysis on anxiety among medical and surgical patients in Ethiopia.

List of figures_202307170548-2.tif This is a preview of your figure rendered on a simulated PLOS journal page.

Maecenas ac est sit amet odio sollicitudin euismod. In risus odio, convallis a neque ac, varius ultricies arcu. Vestibulum et quam iaculis, ultricies odio et, molestie magna. Suspendisse vehicula purus id turpis eleifend, et convallis dui dignissim. Praesent tempus elit a metus sollicitudin, sed fringilla nulla porttitor. Nullam in tempus massa. Nunc maximus magna massa, nec volutpat risus rhoncus ut. Fusce quis ante sem. Aenean nulla nibh, tempus sit amet rhoncus at, eleifend vel risus. Sed dictum, sem ultrices elementum pharetra, lacus diam volutpat orci, scelerisque semper dui lacus ut enim.

Suspendisse in nunc id lacus commodo consequat. Proin semper aliquam varius. Fusce vitae neque aliquam nisi ultrices sodales vitae ut enim. Vivamus nec dictum ipsum. Sed condimentum ante eu urna tincidunt tincidunt. In ac lacus nec ipsum viverra volutpat posuere vel lacus. Class aptent taciti sociosqu ad litora torquent per conubia nostra, per inceptos himenaeos. Morbi rhoncus ipsum quis lorem hendrerit, at vulputate massa tempus. Ut arcu nisl, gravida vitae risus ultricies, porta venenatis massa. Cras dignissim, enim at faucibus aliquam, sapien nisl eleifend dolor, vel mollis nulla nisi id ipsum. Pellentesque vehicula ultricies risus sit amet faucibus. Praesent sit amet mi ac est faucibus accumsan. Praesent pulvinar sit amet orci auctor feugiat.

Phasellus vitae congue est. Duis rutrum iaculis nunc, sed sollicitudin neque eleifend nec. Pellentesque ac nisi eget tortor imperdiet sagittis ut in orci. Mauris porta convallis euismod. Donec in ultricies urna, nec interdum lectus. Nullam sit amet finibus augue, eget rutrum metus. Nam faucibus, urna ac finibus eleifend, neque nisi lobortis ante, at pharetra purus purus sed urna. Curabitur sit amet dui at enim porta posuere non vehicula ligula. Suspendisse potenti. Vestibulum arcu magna, vulputate a massa ac, molestie tincidunt dui.

Donec id tempus lacus, sed tristique nulla. Nullam rutrum risus ut pharetra porttitor. Nam mattis dolor erat, sed volutpat est mattis sed. Suspendisse eu porta tellus. Cras gravida velit sed maximus fermentum. Fusce vitae metus commodo, sagittis nunc sed, faucibus nunc. Integer iaculis quam mattis, luctus neque in, viverra magna. Nulla rhoncus feugiat orci, quis posuere ligula ornare at. Integer vel sagittis risus. Donec semper metus nec finibus accumsan. Mauris sit amet suscipit ante. Aliquam accumsan, nisl vitae vulputate elementum, turpis nibh varius urna, vel bibendum nulla nunc ac quam. Aenean malesuada egestas maximus. Pellentesque faucibus, odio at tincidunt ullamcorper, eros nisi pellentesque mi, non blandit sapien neque quis lectus.

Lorem Ipsum

Lorem ipsum dolor sit amet, consectetur adipiscing elit. Mauris maximus fringilla ligula, in malesuada erat tempor ac. Quisque dapibus posuere turpis, vel aliquam massa vehicula non.

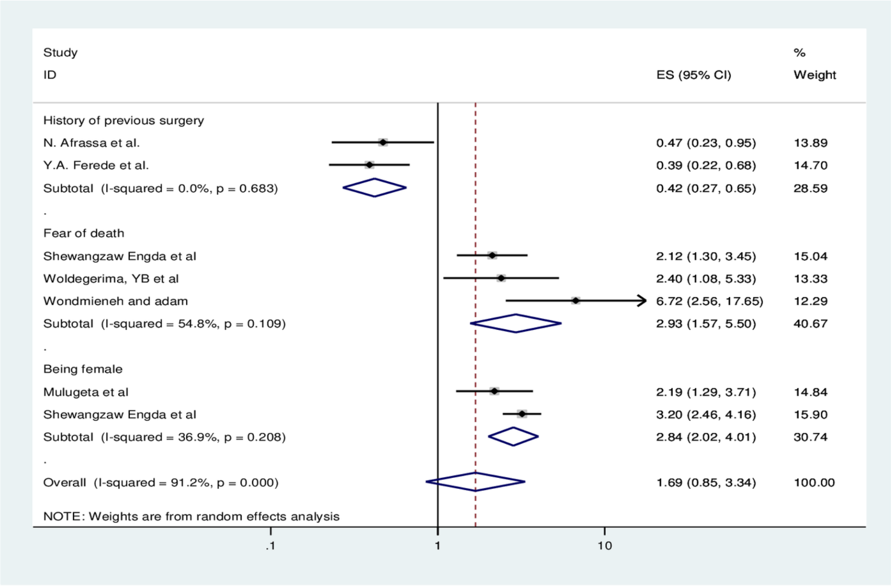

Figure 5: The Forest plot showing factors associated with anxiety among surgical patients in Ethiopia

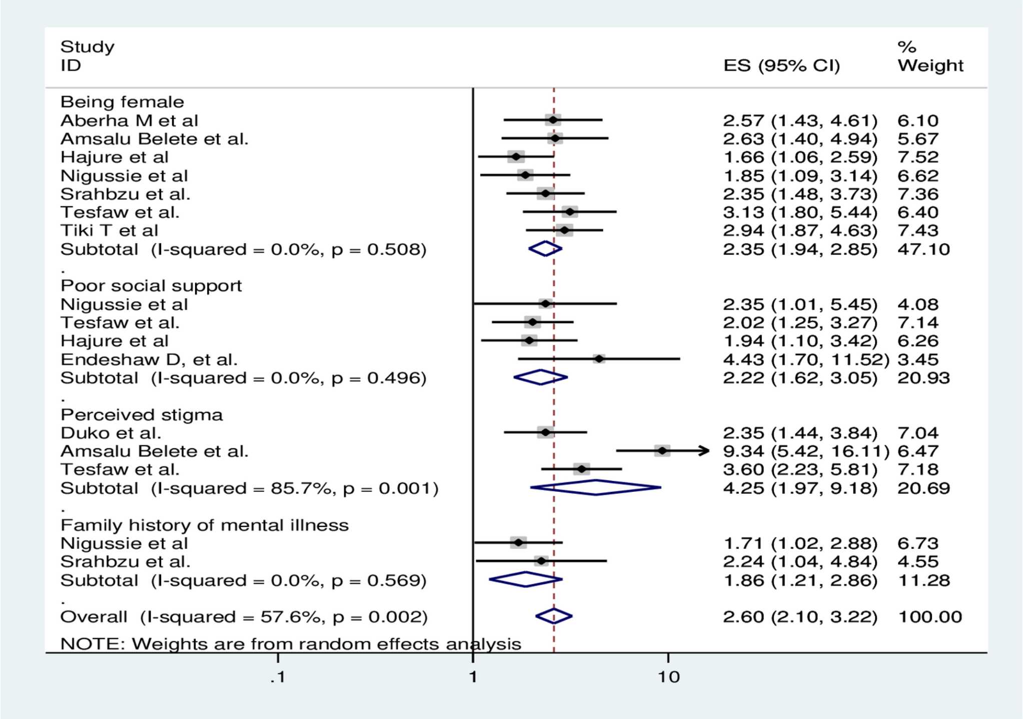

Figure 6: The Forest plot showing factors associated with anxiety among medical patients in Ethiopia.

test-test-1 This is a preview of your figure rendered on a simulated PLOS journal page.

Maecenas ac est sit amet odio sollicitudin euismod. In risus odio, convallis a neque ac, varius ultricies arcu. Vestibulum et quam iaculis, ultricies odio et, molestie magna. Suspendisse vehicula purus id turpis eleifend, et convallis dui dignissim. Praesent tempus elit a metus sollicitudin, sed fringilla nulla porttitor. Nullam in tempus massa. Nunc maximus magna massa, nec volutpat risus rhoncus ut. Fusce quis ante sem. Aenean nulla nibh, tempus sit amet rhoncus at, eleifend vel risus. Sed dictum, sem ultrices elementum pharetra, lacus diam volutpat orci, scelerisque semper dui lacus ut enim.

Suspendisse in nunc id lacus commodo consequat. Proin semper aliquam varius. Fusce vitae neque aliquam nisi ultrices sodales vitae ut enim. Vivamus nec dictum ipsum. Sed condimentum ante eu urna tincidunt tincidunt. In ac lacus nec ipsum viverra volutpat posuere vel lacus. Class aptent taciti sociosqu ad litora torquent per conubia nostra, per inceptos himenaeos. Morbi rhoncus ipsum quis lorem hendrerit, at vulputate massa tempus. Ut arcu nisl, gravida vitae risus ultricies, porta venenatis massa. Cras dignissim, enim at faucibus aliquam, sapien nisl eleifend dolor, vel mollis nulla nisi id ipsum. Pellentesque vehicula ultricies risus sit amet faucibus. Praesent sit amet mi ac est faucibus accumsan. Praesent pulvinar sit amet orci auctor feugiat.

Click here to access/download
Supporting Information
Table of Quality assessment score - anxiety amg M&S pts.docx

extracted data

Click here to access/download
Supporting Information
anxiety on Medical & surgical extracted data F.xlsx

prisma

Click here to access/download
Supporting Information
PRISMA 2020 for anxiety.docx
