# Supplementary material for: Anxiety and associated factors among medical and surgical patients in Ethiopia: A systematic review and meta-analysis
Source: PLoS One. 2024 Jul 24;19(7):e0306413. doi: 10.1371/journal.pone.0306413 (PMC11268606; doi:10.1371/journal.pone.0306413)
Supplement: Attachment — Submitted filename: Point by point response to reviewers comments.docx [file pone.0306413.s005.docx]

**Point by point response to reviewers’ comments**

**Manuscript ID:** **PONE-D-23-22543**

First of all, the authors would like to thank the editor and reviewers for taking their priceless time and giving us crucial feedback/comments throughout the whole manuscript. Accordingly, we have considered all the feedback/comments very important and tried to address them in a better way to improve the quality of the paper. Below, we presented our point-by-point responses to the reviewers’ comments, and the amendments are **highlighted with the track changed** in the revised manuscript. If there is any concern, the authors are very happy to accept and modify it accordingly.

**Reviewers’ comments and responses**

**Reviewer #1:** I have reviewed your manuscript and would like to provide some additional comments and raise concerns regarding potential dual publication.
Firstly, I want to commend you on the thoroughness and clarity of your work. Your research presents valuable insights into [insert topic]. The methodology is well-described, and the results are both robust and significant. Your manuscript has the potential to make a significant contribution to the field.
However, I noticed that there may be some overlap with another publication or work. It's crucial to ensure that your manuscript does not violate the principles of dual publication. Dual publication occurs when substantial parts of a manuscript are published in more than one journal, which can lead to issues such as copyright infringement and academic misconduct.

**Reply:** The authors are grateful for the reviewer’s constructive comments and concerns of dual publication. In the revised manuscript necessary amendments and detail paraphrasing is made. In order to prevent overlaps with others work and committing academic misconduct the authors took the feedback seriously and corrected it accordingly.

**Reviewer #2:** Good attempt at a systematic review but requires some refining. Please address comments added to the document.
Some areas need to be worked on include the summarizing of the abstract and a more detailed and clear results sections (with clear indications of tables and figures).

**Reply:** Thank you for your important suggestion. The abstract section is rewritten in a concise and summarized in an understandable way considering the word limits of the journal guideline. Furthermore, indication of tables and figures are also modified with respect to their text explanations. All the amendments are made in the revised manuscript with highlights of track changed.
