# Supplementary material for: Anxiety and associated factors among medical and surgical patients in Ethiopia: A systematic review and meta-analysis
Source: PLoS One. 2024 Jul 24;19(7):e0306413. doi: 10.1371/journal.pone.0306413 (PMC11268606; doi:10.1371/journal.pone.0306413)
Supplement: Attachment — Submitted filename: Response to reviewers comments33.docx [file pone.0306413.s006.docx]

**Responses to editor and/ or reviewers’ comments**

**Manuscript Title:** Anxiety and associated factors among medical and surgical patients in Ethiopia: A systematic review and meta-analysis
**Manuscript ID:** PONE-D-23-22543

The authors would like to express their gratitude to the editor and reviewers for their invaluable efforts to provide us with useful input and suggestions throughout the article. As a result, we have considered every suggestion, and have tried to better address them to raise the caliber of the work. The points we addressed in our detailed responses to the reviewers' feedback are listed below; the changes are indicated by the track changes in the updated manuscript. Additional language edition and clear explanation about data availability supporting the finding mentioned in the updated manuscript.

**Reviewer #1:**

It should come as no surprise that many different professional associations, government agencies, and universities have adopted specific codes, rules, and policies relating to research ethics. Many government agencies have ethics rules for funded researchers.

**Reply:** It’s obvious that different institutions have their own rule and ethical considerations regarding research ethics specifically when it is funded. However, when it comes to this study it doesn’t create any confusion as it’s a systematic review and meta-analysis. Still, we are thankful for your concern.

**Reviewer #3**

The present study sought to determine the anxiety and associated factors among medical and surgical patients in Ethiopia. The authors were very successful in choosing the research problem, study design, method and articulation of results with the literature. The work is clear, objective, detailed and as a reader, there are no doubts about the theoretical basis, method or how the data were analyzed. The work clearly makes a contribution to the literature, its reading is fluid, and the reader finds it easy to reproduce its detailed method.

**Reply:** Thank you for the positive feedback and the authors are grateful for your genuine comments. We appreciate for sharing us your time and your dedications in commenting our article.
